# A strategy lacking policy?

**DOI:** 10.1590/0102-311XEN020026

**Published:** 2026-06-22

**Authors:** Luciano Bezerra Gomes

**Affiliations:** 1 Centro de Ciências Médicas, Universidade Federal da Paraíba, João Pessoa, Brasil.

The article *Thirty Years of the Family Health Strategy in the Brazilian Unified National Health System: Milestones, Advances, Setbacks and Challenges* ^1^ brings together authors from complementary fields of academic research, all of whom have contributed in different ways to the development of the Family Health Strategy (FHS). The manuscript has several merits that warrant highlighting, including: (1) it provides a clear synthesis of a long and complex trajectory; (2) it brings together evidence showing the improvements in population health brought about by the expansion of access to care; (3) it supports and substantiates criticism of the setbacks triggered by the 2016 coup against President Dilma Rousseff, particularly those occurring during the Jair Messias Bolsonaro administration; (4) it emphasizes positive developments resulting from the resumption of investment and political reorganization in 2023, during Luiz Inácio Lula da Silva third term; and (5) It highlights the critical challenges currently facing primary care including underfunding and poor working terms and conditions and poor working terms.

However, the necessary omissions made due to limited space to be able to make sense of the 30-year trajectory of such a multifaceted area as primary care - especially in a country the size of Brazil and with such a complex health care history - ultimately mean that certain aspects remained underexplored. Or, by opting for a certain theoretical approach or a specific delimitation of what to bring into focus and shed light on, they fail to explore other possibilities that would allow for the construction of divergent readings or, at least, interpretations that create tension. Thus, I take this opportunity to point out some elements that come to mind, after having had the privilege of reading the article firsthand.

First, I would like to revisit the statement made at the beginning of the article in which the authors note that primary care in Brazil “*has witnessed a qualitative leap forward, starting in 1994*” ^1^ (p. 2), following the implementation of what we have for several years referred to as the FHS. I believe this is undeniable. However, they go on to say that this policy “*marked an inflection point for Brazil’s healthcare model, with the adoption of a community-oriented, territorialized approach integrated into the health network and implemented by multidisciplinary health teams*” ^1^ (p. 2). In my view, this is a central issue that requires closer scrutiny, to help us think about how we should use this article to foster debate in Public Health. The suggestion that the policy was “*an inflection point*” and the list of dimensions that underwent change reminded me of an article published in *Cadernos de Saúde Pública* by Luiz Carlos de Oliveira Cecilio & Ademar Arthur Chioro dos Reis in 2018 ^2^. One of the observations made by the authors was the “*disconnect or mismatch between the policy’s wording and its real-life implementation*” ^2^ (p. 1). They go on to wonder “*what actually happens in Brazil’s immense, complex, heterogeneous network of basic care in terms of fulfilling the PNAB*” ^2^ (p. 6), raising the issue that certain elements of the Brazilian National Primary Health Care Policy (PNAB, acronym in Portuguese) framework centralized by the Brazilian Ministry of Health do not materialize in the concrete reality of a significant portion of the country’s services and municipalities. To a large extent, the authors revisit the issue raised by Luiz Cecilio in 1997 ^3^, when he questioned the limitations of overly centralized technocratic approaches centered on the pyramid organizational health service structure - which is at odds with the reality of a significant portion of the population and health workers. In other words, the first question I would raise is the need to examine whether, and if so, to what extent, the FHS has effectively upheld the principles outlined by the authors of the article “Thirty years of the FHS in the SUS” ^1^; and whether it truly marked an inflection point for the care model, or whether we are merely repeating a mantra, projecting our desire for what it does not yet have the power to achieve, despite its potential to do so.

Having established this initial point, I would like to emphasize that the manuscript under discussion acknowledges that “*the FHS has yet to realize its full potential*” ^1^ (p. 2), suffering a number of setbacks under successive governments between 2016 and 2022. The authors ^1^ (p. 2) begin by mentioning “*concerns regarding the future and prospects for this globally recognized innovative PHC model*” and set out to reflect “*the main challenges in achieving its full implementation*”, listing several underlying principles. I refer to this point not in agreement or disagreement over the challenges and principles mentioned by the authors (although I would tend to agree with them), but to take a step back and ask myself: can the FHS really be considered a model? Is there a framework that constitutes an implementable technology-based care model, or is the FHS still a contested concept?

The authors take a clear stance when, at different points throughout the article and in various ways, they suggest that one of the central problems is the uneven implementation of the FHS, indicating that it exists. They go on to outline the model and how it developed by presenting a historical reconstruction of ordinances and laws (Basic Operating Standard - NOB [acronym in Portuguese], PNAB, oral health policy...), highlighting that “*federal policy guidance and financial incentives brought about significant changes in the health care*” ^1^ (p. 6) . They even cite evidence to support this idea, including data illustrating the expansion of physical infrastructure and the number of health teams; however, in my view, they fail to demonstrate that there has been actual change in the technology-based care model. In his PhD dissertation examining the relationship between policymakers and decision-makers in the implementation of public policy, Emerson Elias Merhy ^4^ showed that: (1) it is not the best proposals - those which have won the policymaker debates - that are chosen by decision-makers; (2) it is the policymakers whose political vision and agenda align with the political interests of decision-makers (which encompass but extend beyond health) who are chosen, based on this vision and agenda, to formulate the proposals that will be implemented.

Merhy ^4^ urges us to consider the centrality of the political dimension in the formulation of technology-based care models and the disputes surrounding their implementation. This dimension lies beyond the scope of the excellent essay we have been gifted. To better understand the limitations and challenges involved in implementing the FHS over the first 30 years, we need to ask ourselves to what extent it actually constitutes a technology-based care model (alas there is not enough space left to substantiate my impression that it does not, based on criteria proposed by Merhy ^4^). More than that, we also need to focus not on the implementation of the policy guidelines in place but the process of dispute surrounding policy formulation. It is as if we are dealing with a strategy that does not identify as a policy.

## Data Availability

Not applicable.
