# Supplementary material for: A strategy lacking policy?
Source: Cad Saude Publica. 2026 Jun 22;42:e00020026. doi: 10.1590/0102-311XEN020026 (PMC13286397; doi:10.1590/0102-311XEN020026)
Supplement: Translation

# Translation: Uma estratégia com pouca política?

**DOI:** 10.1590/0102-311XPT020026

**Authors:** Luciano Bezerra Gomes

**Affiliations:** 1 Centro de Ciências Médicas, Universidade Federal da Paraíba, João Pessoa, Brasil.

O artigo *30 Anos da Estratégia Saúde da Família no Sistema Único de Saúde: Marcos, Avanços, Retrocessos e Desafios* ^1^ agrega autores de vertentes complementares da produção acadêmica, todos participantes, em boa medida e de diferentes maneiras, da produção da Estratégia Saúde da Família (ESF) que aqui analisam. Este manuscrito apresenta diversos méritos que precisam ser salientados, dentre os quais, destaco: (1) elabora boas sínteses de uma longa e complexa trajetória; (2) consolida evidências sobre a melhoria das condições de saúde da população em virtude da expansão do acesso; (3) sustenta e embasa posição crítica sobre os retrocessos desencadeados após o golpe de 2016, sobre a Presidenta Dilma Rousseff, e especialmente no governo Jair Messias Bolsonaro; (4) sinaliza aspectos positivos ocorridos com a retomada de investimentos e reorganização política a partir de 2023, pelo terceiro governo Luiz Inácio Lula da Silva; (5) aponta desafios fundamentais para a atenção básica na atualidade, como financiamento insuficiente, precarização de vínculos de trabalho, entre outros.

Não obstante, os recortes que se fazem necessários para, num espaço tão curto como de um artigo, construir inteligibilidade sobre uma trajetória de 30 anos em torno de uma área tão múltipla como a atenção básica, num país com as dimensões - e o histórico na saúde - do Brasil, acabam levando à insuficiência na exploração de certas dimensões. Ou fazem com que, ao optar por certa abordagem teórica ou determinada delimitação do que trazer para a lente e lançar luzes, deixa de explorar outras possibilidades, que permitiriam construir leituras divergentes ou, pelo menos, que se tensionam. Assim, aproveito o espaço para apontar alguns elementos que me saltaram à mente, dado o privilégio da leitura em primeira mão do artigo.

Em primeiro lugar, retomo uma afirmação logo do início deste ensaio, em que os autores constatam que a atenção básica no Brasil “*teve um salto qualitativo a partir de 1994*” ^1^ (p. 2), desde a implementação do que, há vários anos, denominamos como Estratégia Saúde da Família (ESF), e creio que isso é inegável. Porém, logo em seguida, eles afirmam que tal política “*marcou uma inflexão no modelo assistencial, com orientação comunitária, territorializada, integrada à rede e equipe multiprofissional*” ^1^ (p. 2) e isso, a meu ver, é uma questão central sobre a qual precisamos colocar uma lupa, para pensarmos em como que devemos aproveitar esse artigo para fomentar o debate na Saúde Coletiva. Ao indicar que se promoveu uma “*inflexão no modelo assistencial*” e listar várias dimensões que teriam sido modificadas, remeti-me a um artigo publicado em 2018 em *Cadernos de Saúde Pública*, por Luiz Carlos de Oliveira Cecilio & Ademar Arthur Chioro dos Reis ^2^. Dentre os apontamentos que fazem, os autores destacam a “*disjunção ou o descompasso entre a formulação da política e sua implementação real*” ^2^ (p. 1) e se perguntam sobre “*o que acontece, de fato, na imensa, complexa, heterogênea rede de atenção básica brasileira em termos de realização da PNAB*” ^2^ (p. 6), problematizando que elementos da formulação centralizada pelo Ministério da Saúde em torno da Política Nacional de Atenção Básica (PNAB), não se materializam na vida concreta de boa parte da realidade dos serviços e municípios do país. Em boa medida, estes autores retomam uma provocação que o próprio Luiz Cecilio havia feito em 1997 ^3^, quando questionou as limitações de formulações tecnocráticas excessivamente centralizadas, em torno do modelo piramidal proposto para estruturar os serviços de saúde, em desacordo com a vida de boa parte das populações e trabalhadores nos serviços de saúde. Essa primeira questão que preciso colocar, em outras palavras, refere-se à necessidade de problematizar se e em que medida a ESF efetivamente materializou os princípios apontados pelos autores do artigo “30 anos da ESF no SUS” ^1^, se levou mesmo a uma inflexão no modelo assistencial; ou se apenas repetimos como mantra projetando nosso desejo do que ela ainda não tem potência, embora tenha potencial.

Posta essa primeira pauta, saliento que o próprio manuscrito ora em debate reconhece que “*a ESF não se desenvolveu em sua plenitude*” ^1^ (p. 2) , além de ter sofrido com os retrocessos dos governos entre 2016 e 2022. E assim, os autores ^1^ (p. 2) partem da “*inquietação sobre o futuro e perspectivas deste modelo inovador*” e propõem-se a pensar “*sobre os principais desafios para sua plena implementação*”, listando diversos princípios constitutivos. E me remeto a isso, não para concordar ou discordar dos desafios e princípios que eles apontam (se o fizesse, minha tendência seria de alinhamento com eles), mas em dar um passo para trás e me perguntar: a ESF pode mesmo ser tomada como um modelo? Haveria tal formulação delineada de maneira a constituir um projeto tecnoassistencial a ser implementado, ou teríamos que considerar que a ESF é uma elaboração ainda em disputa.

Os autores se colocam num polo definido ao se referirem, em diferentes momentos e de modos variados, à ideia de que um dos problemas centrais seria a implementação heterogênea da ESF, se remetendo a tal modelo como existindo. E quando delineiam o que seria tal modelo e como ele foi se constituindo, o fazem por meio da reconstrução histórica das portarias e normativas (Norma Operacional Básica - NOB, PNAB, Política de Saúde Bucal...), por considerar que as “*diretrizes políticas e os incentivos financeiros federais promoveram mudanças expressivas no modelo assistencial*” ^1^ (p. 6). E usam como elementos para fundamentar tal concepção a evolução positiva de dados de expansão de estrutura física, de quantidades e modalidades de arranjos de composição de equipes, entre outros dados positivos, mas não conseguem demonstrar, a meu ver, a ocorrência de efetiva modificação do modelo tecnoassistencial. Emerson Elias Merhy ^4^ já nos ensinara, ao estudar em sua tese de doutorado como se dá a relação entre formuladores e decisores na implementação das políticas públicas, que: (1) não são as propostas demonstradas como melhores que, ao vencer a discussão entre os formuladores, são escolhidas pelos decisores; (2) são os formuladores cujas visões e projetos políticos se aproximam mais dos interesses políticos dos decisores (para além da saúde, mas que passam por ela) aqueles que são escolhidos para, a partir disso, formularem as propostas que serão implementadas.

O que Merhy ^4^ provoca a pensar é a centralidade da dimensão política em torno na formulação e das disputas pela implementação dos modelos tecnoassistenciais. E em alguma medida, essa dimensão escapa do excelente ensaio que estamos recebendo de presente. Compreender melhor os limites e desafios da implementação da ESF em seus 30 anos requer, em minha opinião, pensar o quanto ela constitui efetivamente um modelo tecnoassistencial (e o espaço aqui é insuficiente para fundamentar, com base nos critérios propostos por Merhy ^4^ que minha impressão seria que não se configura como tal) e, mais que isso, trazer para o centro do foco não a implementação das diretrizes formuladas, mas o próprio processo de disputa em torno das suas formulações. É como se estivéssemos diante de uma estratégia que não se reconhece como política.

## Data Availability

Não se aplica.
